# Assessing constraints on the path of regulatory sequence evolution

**DOI:** 10.1098/rstb.2013.0026

**Published:** 2013-12-19

**Authors:** William J. Glassford, Mark Rebeiz

**Affiliations:** Department of Biological Sciences, University of Pittsburgh, 4249 Fifth Avenue, Pittsburgh, PA 15260, USA

**Keywords:** *cis*-regulatory evolution, mutational path, sign epistasis, pleiotropy

## Abstract

Structural and functional constraints are known to play a major role in restricting the path of evolution of protein activities. However, constraints acting on evolving transcriptional regulatory sequences, e.g. enhancers, are largely unknown. Recently, we elucidated how a novel expression pattern of the *Neprilysin-1* (*Nep1*) gene in the optic lobe of *Drosophila santomea* evolved via co-option of existing enhancer activities. *Drosophila santomea*, which has diverged from *Drosophila yakuba* by approximately 400 000 years has accumulated four fixed mutations that each contribute to the full activity of this enhancer. Recreating and testing the optic lobe enhancer of the ancestor of *D. santomea* and *D. yakuba* revealed that the strong *D. santomea* enhancer activity evolved from a weak ancestral activity. Because each mutation on the path from the *D. yakuba/santomea* ancestor to modern-day *D. santomea* contributes to the newly derived optic lobe enhancer activity, we sought here to use this system to study the path of evolution of enhancer sequences. We inferred likely paths of evolution of this enhancer by observing the transcriptional output of all possible intermediate steps between the ancestral *D. yakuba/santomea* enhancer and the modern *D. santomea* enhancer. Many possible paths had epistatic and cooperative effects. Furthermore, we found that several paths significantly increased ectopic transcriptional activity or affected existing enhancer activities from which the novel activity was co-opted. We suggest that these attributes highlight constraints that guide the path of evolution of enhancers.

## 1. Introduction

Evolution often proceeds through the accumulation of numerous mutations that collectively generate meaningful phenotypic outcomes [1–5]. The order in which such changes are introduced may differ substantially in the functional consequences of intermediates. Although much attention has been focused on the path by which proteins evolve [6–8], the constraints and complications that arise during the multi-step evolution of non-coding transcriptional activating sequences (enhancers) are less understood. Moreover, regulatory DNA has become increasingly appreciated as a major source of phenotypically relevant variation, particularly contributing to the evolution of morphology [4,9–12].

Although enhancers are frequently conserved [13–17], they often diverge more rapidly than protein-coding sequences [18–20]. This is, in part, due to the constraints that the triplet amino acid code imposes on protein-coding DNA. Enhancers contain assemblages of docking sites for transcription factors that collectively influence the initiation rate of transcription [21]. Great amounts of variation can be observed in the presence, spacing and sequence of transcription factor binding sites within and between species, often resulting in regulatory sequences that maintain function despite extreme sequence variation [22–24]. Therefore, in order to assess how an enhancer might accumulate a number of functionally relevant changes, one must look to either slowly evolving regions, or at differences that have arisen over short evolutionary periods.

### (a) Possible constraints on the evolution of regulatory DNA

There are several possible constraints that may disfavour certain mutational paths of regulatory sequences. These may include the preservation and improvement of the evolving activity, the maintenance of pre-existing functions and the context dependence of mutations [2,25]. In the case of adaptive evolution, driven by constant directional selection, it is generally accepted that an evolving protein-coding or regulatory DNA must improve, or not diminish, the fitness of the organism with each step [26]. This constant refinement of a derived activity can be constrained by epistatic and pleiotropic interactions.

Pleiotropy, the effect of a single mutation on multiple traits, appears to be a major constraint on evolutionary paths [27]. Although the pleiotropic consequences of mutations to regulatory DNA are predicted to be milder compared with protein-coding regions [28,29], individual mutations may nonetheless lead to context-specific pleiotropic consequences. These may involve effects on other expression patterns of the gene in question, alteration of the regulation of adjacent genes [30] or the occurrence of unwanted ectopic expression. However, these pleiotropic effects may be circumvented by transitions that include epistatic interactions.

Epistasis, the dependence of a mutation's effect on the genetic background, could cause a path to be less favoured compared with other paths that successively increase expression. An extreme case of epistasis, sign epistasis [31], generates opposite effects of a mutation in different backgrounds. In a system under strong positive directional selection, where each step must increase an activity, paths that exhibit sign epistasis would be strongly disfavoured. During the course of protein evolution, sign epistasis often restricts evolutionary trajectories that pass through structurally unstable intermediate states [6,8]. However, regulatory sequences have been posited to be less susceptible to such destabilizing mutations [4].

Robustness, the generation of reproducible outcomes in response to a highly varied environment, has been a topic of much recent interest in the field of regulatory biology. For example, the maintenance of robustness has been cited as a cause for the existence of ‘shadow enhancers’ [32,33]—the phenomenon that often multiple enhancers exist for a similar activity in the same gene [34]. In two separate instances, the removal of a shadow enhancer while maintaining the other copy has caused a decrease in robustness: animals lacking the ‘shadow’ copy show greater variability in phenotype when grown at differing temperatures or in differing genetic backgrounds [32,33]. It has also been shown that apparently redundant binding sites within a single enhancer may be required to foster robustness [35]. It is generally assumed that the establishment of robustness is a common step during an enhancer's evolution. However, we currently lack examples that demonstrate an enhancer evolving from a less robust state into a more robust state.

### (b) A model for studying the path of regulatory evolution

While the constraints on a regulatory sequence's evolution can be easily imagined, we currently lack fundamental knowledge of what is possible during an enhancer's path of evolution. How pervasive is epistasis? What kinds of epistatic interactions exist? When and how can robustness evolve? What other unexpected constraints on enhancer evolution exist? Given the prevalence and rapidity of regulatory DNA evolution, the identification of forces constraining evolutionary paths represents an important step in understanding how regulatory sequences acquire altered functions.

**Figure 1.**
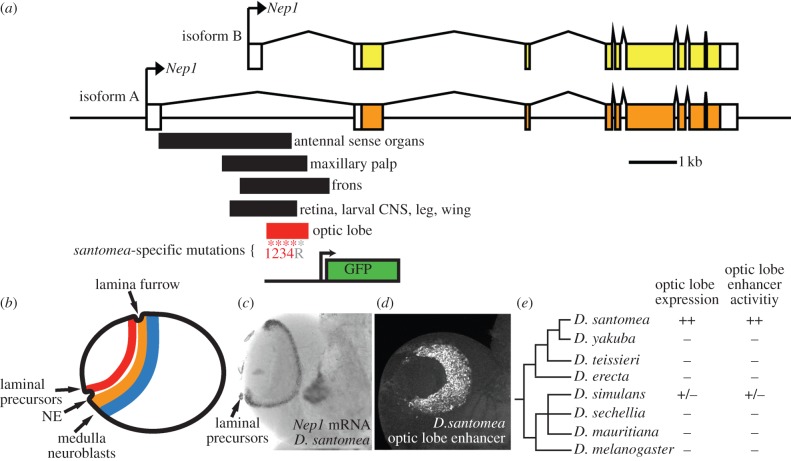
A recently evolved expression pattern of the *Nep1* gene depends upon four mutations fixed in *Drosophila santomea*. (*a*) The first *Nep1* intron contains several overlapping transcriptional enhancers, including an optic lobe enhancer novel to *D. santomea*. The *D. santomea* enhancer differs from the derived *D. yakuba/santomea* ancestral optic lobe enhancer by four mutations, marked by red asterisks. The position of a polymorphic repeat expansion in modern-day *D. santomea* is marked by a grey asterisk. The extent of a GFP reporter construct used in this study is denoted. (*b*) *Nep1* optic lobe activity occurs in laminal precursors, a transition state that occurs as neuroepithelial (NE) cells migrate past the lamina furrow and become lamina neurons. NE cells that migrate away from the furrow transition to medulla neuroblasts. (*c*) *In situ* hybridization of the *D. santomea* third-instar larval optic lobe with a *Nep1* riboprobe reveals *Nep1* expression in lamia precursor cells (arrow) and the mushroom body. (*d*) The *D. santomea* optical lobe enhancer reporter construct drives expression in lamina neurons. (*e*) Phylogeny of *D. yakuba* and *D. melanogaster* clades referencing optic lobe expression assayed via *in situ* hybridization and by species-specific reporter constructs. Plus symbols denote strong expression, minuses denote absence of expression, plus/minus symbol denotes weak expression [36].

Recently, we elucidated the origins of a newly evolved enhancer activity that arose in the *Nep1* gene of *D. santomea* [36]. Optic lobe expression of *Nep1* in lamina precursors (figure 1*b*,*c*) is unique to the *D. santomea* visual system. This novel expression pattern is encoded by a 680 bp enhancer element embedded in the first intron of the *Nep1* gene (figure 1*a*,*d*). The novel optic lobe activity of *D. santomea Nep1* overlaps several other enhancer regions in the intron, suggesting that perhaps this activity sprouted out of a pre-existing adjacent enhancer (figure 1*a*). In a series of mutant reporters, we determined that the novel optic lobe activity depends upon short stretches of nucleotides required for full activity of other ancestral overlapping activities in the retinal field and central nervous system (CNS). Further, by sequencing this segment from multiple (at least 14) isofemale lines of *D. yakuba, D. santomea* and the closest outgroup *Drosophila teissieri*, we found that the *D. santomea* optic lobe enhancer differs from the *D. yakuba/santomea* ancestor by just four fixed mutations. In an *in vivo* reporter assay, we found that reversion of each of these mutations in the context of the *D. santomea* enhancer led to a significant reduction in activity. By reverting all four of these mutations simultaneously, we tested the activity of the resurrected *D. yakuba/santomea* ancestral enhancer. We found that this enhancer had a weak activity in the optic lobe, suggesting that this was the starting point for the strong, derived optic lobe expression of *D. santomea Nep1*. Although it is uncertain whether the changes at *Nep1* were adaptive, its novel optic lobe activity is unique in that it is an experimentally tractable example in which a short path of mutations leads to greatly increased enhancer activity.

Here, we use the recently evolved optic lobe activity of *Nep1* to assess constraints that may influence the path of an enhancer's evolution. From the starting point of the reconstructed *D. yakuba/santomea* ancestral enhancer sequence, we tested each possible evolutionary intermediate in an *in vivo* reporter assay in order to identify ways in which the order of introduction may be restricted. First, we observe sign epistasis: introduction of certain mutations can increase or decrease activity depending on the mutational trajectory. Further, we noted that some paths modulate the activities of an overlapping enhancer, which could influence fitness. Finally, we observe paths which progress through intermediates with strong ectopic activity that manifest under chronic temperature stress. These results provide empirical evidence of the types of constraints that are likely to influence the ordering of mutations that are acceptable during the diversification of regulatory sequences under persistent, directional selection for increased expression.

## 2. Material and methods

### (a) Transgenic constructs

Mutated versions of the *Nep1* enhancer fragment were produced by overlap extension PCR using primer sequences described previously [36]. Constructs differ only at the noted sites; the entire sequence of each construct was confirmed via sequencing. PCR products were cloned into the S3aG transgenesis plasmid [37] using *Asc I* and *Sbf I* sites. S3aG contains a multi-cloning site upstream of a basal promoter driving enhanced nuclear green fluorescent protein (GFP) derived from the pH-Stinger series of vectors [38] as well as a donor attB site for site-specific insertion into the *Drosophila melanogaster* genome. Constructs were injected by Rainbow Transgenic Flies (Camarillo, CA) into a *φ*C31-integrase expressing line with an attP insertion on the second chromosome (51D) [39]. Independent transgenic lines were outcrossed to a *yellow–white* stock for two generations before the establishment of homozygous insertions.

### (b) Quantification of reporter activity

Late third-instar female larvae for at least two lines were dissected in cold PBS, and fixed in 4% paraformaldehyde PBT (PBS + 0.1% Triton-X-100) solution for 30 min at room temperature. Samples were washed several times in PBT and then incubated in a 50% glycerol/PBT solution for 10 min before mounting on slides in glycerol mountant (80% glycerol, 0.1 M Tris, pH 8.0).

Mounted brains and imaginal discs were imaged on an Olympus Fluoview 1000 confocal microscope using standardized non-saturated settings. Maximum projections of reporter construct expressing brains were saved, and fluorescent intensity was quantified using the ImageJ software with the freehand selection tool. The region used for intensity measurements was chosen by making selections on duplicated images whose brightness was increased, and subsequently measuring these selections on un-manipulated images. Expression intensity was compared using one-way ANOVA in the JMP-pro software package (SAS Institute, Cary, NC). Post-hoc pairwise comparisons of sample means were carried out with the Tukey's honestly significant difference (HSD) test.

## 3. Results

**Figure 2.**
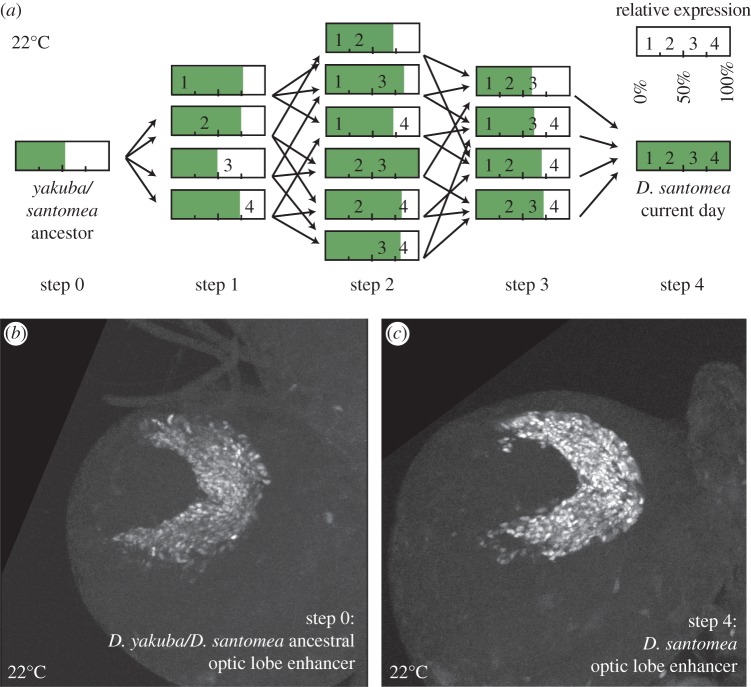
Map of the effects of mutational paths on enhancer activity in the optic lobe. (*a*) Schematic of intermediates between the *Drosophila yakuba/santomea* ancestral optic lobe enhancer and the *D. santomea* modern-day optic lobe enhancer illustrates possible evolutionary pathways. Each bar represents a reporter construct. Numbers within each bar denote the presence of one or more mutations on the path to modern-day *D. santomea.* The shading represents reporter expression quantified from the lamina neurons of third-instar larval brains grown at 22°C, relative to the *D. santomea* construct. (*b*) Optic lobe of *D. yakuba/santomea* ancestral optic lobe enhancer reporter construct. (*c*) Optic lobe of *D. santomea* modern-day optic lobe reporter construct. (Online version in colour.)

To detect possible constraints on the evolutionary ordering of regulatory sequence mutations, we constructed mutant versions of an 1176 bp non-coding DNA segment containing the *D. santomea Nep1* optic lobe enhancer [36]. Working with four mutations that were fixed in the *D. santomea* lineage, we generated and tested all possible combinations of these mutations in the context of this fragment (figure 2*a*). These versions represent all possible evolutionary intermediates along the trajectory from the reconstructed ancestor of *D. yakuba* and *D. santomea* to modern-day *D. santomea.* By inserting these constructs into the same genomic position as in our previous study, we were able to control for positional effects on reporter activity. Previously, the reconstructed ancestor was engineered to remove a repeat expansion that is polymorphic in *D. santomea* [36] (figure 1*a*, ‘R’). To recreate intermediates from the *D. yakuba/santomea* ancestor to the modern-day *D. santomea*, we added this repeat, which represents the most common allele in the sample (4/14 sequences). Doing so resulted in a 22% increase in expression from 30% to 52% relative to the modern-day *D. santomea* construct (see electronic supplementary material, figure S1*a*). Thus, this highly divergent region that is mostly composed of unique alleles in our sequence sample [36] influences the optic lobe activity.

Overall, the activity level of these intermediates varied from low expression similar to the ancestral enhancer (52% of modern-day *D. santomea*) to high activity that resembled the modern-day *D. santomea* construct (table 1 and figure 2*a*). The dataset was analysed by one-way ANOVA followed by pairwise comparisons, using post-hoc Tukey's HSD tests (**α** = 0.05), which statistically accounts and corrects for multiple, simultaneous comparisons. In the following, we report observed constraints that may restrict the path by which this enhancer could have accumulated these four fixed differences.

**Table 1.** Relative expression levels of *Nep1* optic lobe enhancer intermediates.

construct	*N*	relative activity (%)	s.e.m. (%)	class**^a^**				
ancestor	36	52.5	2.5				D	E
ancestor FW1	31	78.8	3.5			C		
ancestor FW2	38	76.6	2.8			C		
ancestor FW3	27	49.2	2.6					E
ancestor FW4	51	72.4	2.0			C		
double Mut 1–2	20	71.5	4.4			C	D	E
double Mut 1–3	16	84.2	6.1	A	B	C		
double Mut 1–4	14	72.7	6.8			C	D	E
double Mut 2–3	55	102.6	3.2	A				
double Mut 2–4	42	81.2	4.6			C		
double Mut 3–4	20	79.1	4.4		B	C		
triple Mut 2–3–4	16	73.0	7.6			C	D	E
triple Mut 1–3–4	16	65.5	8.1			C	D	E
triple Mut 1–2–4	22	72.3	4.0			C	D	
triple Mut 1–2–3	59	55.8	2.4				D	E
santomea	37	100.0	4.9	A	B			

^a^Mutant constructs connected by the same letter are not significantly different (*p* > 0.05).

### (a) Epistatic effects

**Figure 3.**
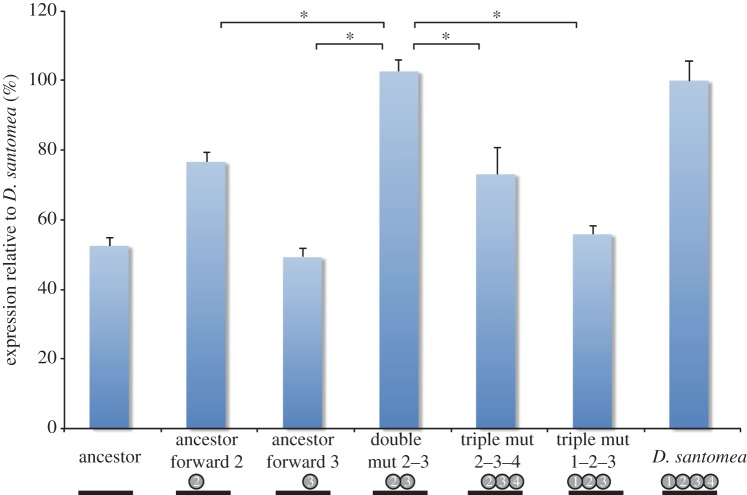
Epistatic non-additive interactions and sign epistasis between mutations mark the path of the *Nep1* enhancer's evolution. While addition of mutation 3 to the ancestral construct does not result in a significantly different activity increase (ancestor forward 3), addition of mutation 2 to this construct results in a drastic increase in activity (double Mut 2–3). Sign epistasis is revealed upon the introduction of mutation 1 (Triple Mut 1–2–3) or mutation 4 (triple Mut 2–3–4) into this background, which both decrease activity. All activity values are normalized to modern-day *D. santomea* levels. Error bars show standard error of the mean. Asterisks denote significant differences (Tukey's HSD test, **α** = 0.05). (Online version in colour.)

Upon reconstructing the possible ways that the four fixed mutations could have accumulated, we noted non-additive interactions between the mutations 2 and 3 (figure 3). Although these mutations had significant effects when removed from *D. santomea* (table 1, triple Mut 1–3–4, triple Mut 1–2–4), mutation 3 showed no significant effect when introduced in the background of the ancestral construct (figure 3, ancestor forward 3). However, when mutation 2 is added to mutation 3 (figure 3, double Mut 2–3), an approximately twofold increase in enhancer activity was observed. Thus, the presence of mutation 2 is required for the effect of mutation 3. Several additional intermediate steps similarly represented ‘lateral moves’ in which a significant increase in activity was not detected. Indeed, of 24 possible paths connecting the ancestor to modern-day *D. santomea*, 22 contained steps that did not significantly increase activity in uncorrected pairwise *t*-tests (**α** = 0.05). Thus, although each mutation is required for modern-day *D. santomea* activity levels, nearly every path includes a transition that does not notably increase expression. Moreover, many paths, including the remaining two of 24, exhibit steps resulting in a significant decrease in expression (discussed further in §3*b*).

### (b) Sign epistasis

In addition to epistatic interactions, we also observed that several mutational paths involved significant sign epistasis (figure 3). For example, the intermediate that combines mutations 2 and 3 has a high activity, 103% of modern-day *D. santomea* (figure 3). Subsequent addition of mutations 1 or 4 leads to a 47% or 30% reduction in activity, respectively (figure 3, ‘triple Mut 2–3–4’, ‘triple Mut 1–2–3’). Of the 24 possible paths, a full six of these include steps that show significant sign epistasis (*p* < 0.05, Tukey's HSD test). Six additional paths suggested sign epistasis, with transitions that were detected as significant decreases in an uncorrected paired *t*-test (*p* < 0.05). Thus, a full half of the possible trajectories connecting the *D. yakuba/santomea* ancestor to modern-day *D. santomea* included significant or suggestive sign epistasis.

### (c) Ectopic expression

**Figure 4.**
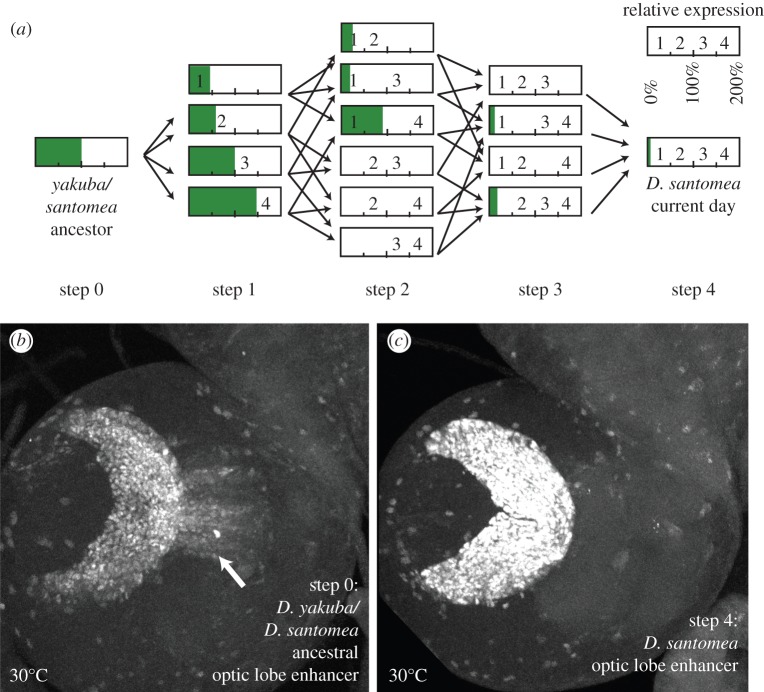
The effects of mutational path on ectopic expression. (*a*) Schematic of intermediates between the *Drosophila yakuba/santomea* ancestral optic lobe enhancer and the *D. santomea* modern-day optic lobe enhancer illustrating possible evolutionary pathways. Each bar represents a reporter construct, and numbers denote which *D. santomea*-specific mutations are present. Shading represents reporter expression quantified from the medullar neuroblasts of third-instar larval brains grown at 30°C, normalized to the level of the *D. yakuba/santomea* ancestral construct. (*b*) Optic lobe of *D. yakuba/santomea* ancestral optic lobe enhancer reporter construct grown at 30°C reveals ectopic expression in a region of the medulla (arrow); (*c*) optic lobe of *D. santomea* modern-day optic lobe reporter construct grown at 30°C lacks this ectopic activity. (Online version in colour.)

During our analysis of the possible evolutionary trajectories, we noted ectopic expression in some paths, manifesting in a zone of the medulla adjacent to the laminal precursors that express *Nep1* in *D. santomea* (figure 4*b*, arrow). This ectopic activity was particularly notable when lines were reared under chronic temperature stress at 30°C. The resurrected ancestor of *D. yakuba* and *D. santomea* had a fairly high level of ectopic expression in this location (figure 4*b*), whereas *D. santomea* exhibited little to no ectopic expression (figure 4*c*). Measuring the ectopic expression of different trajectories at 30°C, we noted that distinct paths increased or decreased this ectopic activity to differing extents (figure 4*a*). For example, addition of mutation 4 to the ancestor led to enhanced ectopic expression (≈ 1.5-fold increase, *p* < 0.05, uncorrected paired *t*-test), whereas introduction of mutation 2 or 3 to ancestor forward 4 completely ablates it. Most paths that did not include the early addition of mutation 4 exhibited the general trend of reducing ectopic activity (figure 4*a*).

### (d) Effects on overlapping activities

**Figure 5.**
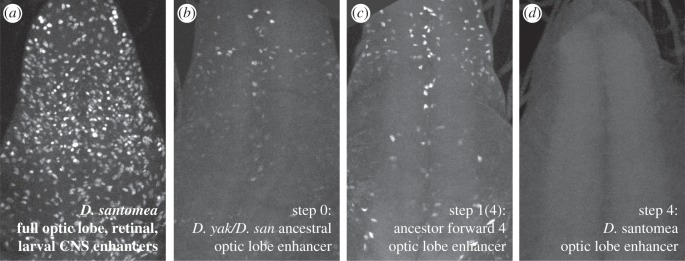
Individual paths differentially contribute activity to a known expression pattern of *Nep1*. (*a*) Expression of a *Nep1 r*eporter construct containing the retinal field, larval CNS and optic lobe enhancers in the ventral ganglion. (*b*–*d*) The *D. yakuba/santomea* ancestral optic lobe enhancer reporter is expressed in a small proportion of the ventral ganglion (*b*), whereas the *D. santomea* optic lobe enhancer drives no expression (*d*). The ancestor forward 4 construct drives increased expression in the ventral ganglion (*c*).

In addition to pleiotropic activation of the reporter in ectopic locations, we observed intermediates that had effects on a different tissue where *Nep1* is deployed: the larval CNS (figure 5*a*). Larval CNS expression exhibited by the ancestral construct (figure 5*b*) is absent in the modern-day *D. santomea* construct (figure 5*d*), and is increased in an intermediate construct, ancestor forward 4 (figure 5*c*). These results suggest that different trajectories would appear to modulate existing CNS activity of *Nep1*, and larval CNS activity is reduced during the evolution of the optical lobe enhancer from the *D. yakuba/santomea* ancestral state. We conclude the path of the *Nep1* optic lobe enhancer's evolution can alter overlapping endogenous functions of the *Nep1* gene that may impact fitness.

## 4. Discussion

Here, we have examined several factors that may commonly restrict a regulatory sequence's path of evolution. By generating and testing a comprehensive set of all possible evolutionary intermediates in an *in vivo* assay, we explored the biological pitfalls of individual mutational paths, and compared their merits. Although each of the four mutations that we characterized increased activity in at least one setting, every single path included non-additive or sign-epistatic legs along the journey to the modern-day *D. santomea Nep1* enhancer. Above and beyond the sign and magnitude of expression differences between intermediates, our findings suggest that not all paths are equal in terms of pleiotropic effects on pre-existing and ectopic activities. Nevertheless, no combination of mutations caused the enhancer to fail utterly. Although we cannot comment on the biological significance of *Nep1* expression in the optic lobe, or what negative fitness consequences would result from paths that induced pleiotropic effects, the constraints that are revealed by our study illuminate what is possible for an evolving regulatory sequence. Indeed, some of the constraints we examined would pertain to both adaptively and neutrally evolving enhancers. These findings provide a more nuanced view of the complexities associated with evolving increased enhancer activity.

### (a) Epistatic interaction and enhancer information processing mechanisms

Under a model of persistent directional selection, epistasis is predicted to constrain potential paths of evolution. Although our initial experiments with the *Nep1* enhancer of *D. santomea* suggested that each of the four fixed mutations are required to generate the full activity of the *D. santomea* enhancer [36], our reconstruction of all possible paths revealed how the process of introducing these mutations in sequence was not straightforward. Indeed, each of the four mutations had contexts in which their addition had no effect on expression level. Mutation 3 presents a very clear case of cooperative interaction (figure 3), as it only increased activity in a limited number of contexts (figure 2). Moreover, the polymorphic repeat expansion (figure 1*a*, ‘R’) introduces additional epistatic interactions (see electronic supplementary material, figure S1), illustrating how polymorphisms could further complicate the interpretation of mutational effects.

In a now highly influential review, Arnosti & Kulkarni [40] put forward two contrasting models of how enhancers process information: billboards and enhanceosomes. In the enhanceosome model, the enhancer DNA acts as a scaffold to form a higher-order conformation of interacting proteins. Such a model is supported by the precise requirement for the presence and spacing of all of the binding sites in the enhancer to activate transcription [41]. By contrast, the billboard model suggests that spacing and cooperative interaction of binding sites is minimal, and that the net output of such an enhancer is the collective interpretation of positive and negative inputs. Although it is well-recognized that enhancers may incorporate aspects of enhanceosome and billboard architecture simultaneously [40], these two contrasting models of enhancer action are predicted to differ in the flexibility of their evolutionary paths. A billboard enhancer that is evolving new binding sites would be predicted to be unconstrained by epistatic effects, whereas an enhancer that follows the enhanceosome model would have many (if not all) paths that include epistasis.

Considering our data in the light of the enhanceosome and billboard models, we suggest that the derived activity of the *D. santomea* optic lobe enhancer of *Nep1* likely represents a combination of both. The widespread epistatic effects we observe are consistent with the evolution of binding sites for proteins that interact physically, as expected of an enhanceosome. However, in none of the intermediates is expression completely lost, or reduced below the level observed for the ancestor. Thus, the aspects of the optic lobe enhancer are consistent with a billboard architecture as well.

### (b) The prevalence and possible mechanisms of sign epistasis

The prevalence of sign epistasis in protein-coding sequences is often attributed to trade-offs between thermodynamic stability and the evolution of new functions [8,6]. In the case of TEM β-lactamase, Weinreich *et al*. [8] observed sign epistasis between a mutation that increases antibiotic hydrolysis while concurrently reducing its stability, with a second mutation that increases thermodynamic stability, but slightly reduces activity. For several of the paths, introduction of the stabilizing mutation was deleterious in the absence of the activity-increasing mutation. By contrast, Ortlund *et al*. [6] found that mutations which increased thermodynamic stability were required before major function-altering mutations could evolve in the vertebrate glucocorticoid receptor. In this case, the mutations to the binding pocket of the protein were so dramatic that unstable intermediates would form in the absence of these permissive mutations. As thermodynamic instability represents a dead end for an evolving protein, sign epistasis is expected to be a rigid constraint during the path of coding sequence evolution.

**Figure 6.**
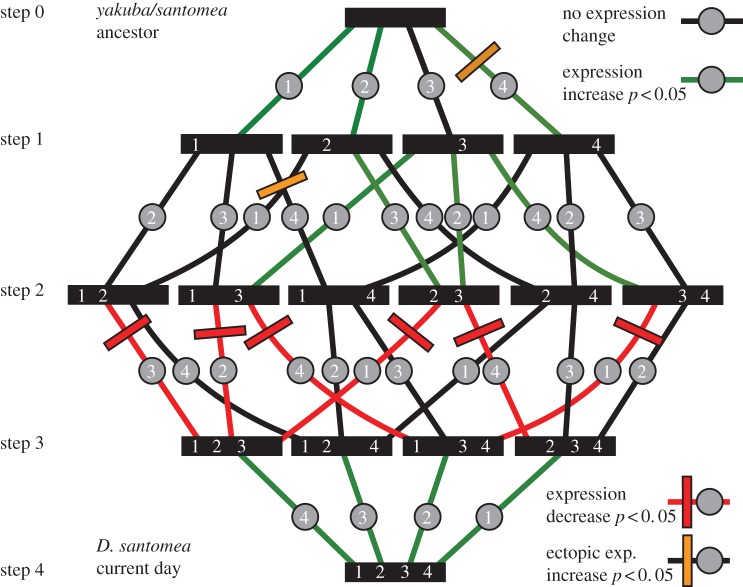
Summary of constraints restricting the path of evolution of the *Nep1* enhancer. This schematic illustrates the constraints that may guide the path of evolution of the *Nep1* enhancer. The black bars with white numbers represent possible mutational intermediates between the *Drosophila yakuba/D. santomea* ancestral enhancer and the modern-day *D. santomea* enhancer. Lines represent possible evolutionary pathways between the ancestral and the modern-day enhancer: green paths represent a significant increase in expression (Tukey's HSD test **α** < 0.05), red paths represent a significant or suggestive decrease in expression (*p* < 0.05) and black paths represent an insignificant change in expression. Coloured bars that cross individual routes represent possible constraints that may reduce the viability of a given evolutionary pathway: red bars are on paths with a suggestive decrease in activity (*p* < 0.05), orange bars are on paths with a suggestive increase in ectopic activity (*p* < 0.05).

An unexpected finding of this work was the frequency of sign epistasis among the reconstructed evolutionary trajectories of a regulatory sequence. Each and every mutation had at least one context in which its introduction would decrease the expression level from a previous step (figure 6). There are several possible explanations for how mutations to an enhancer could generate opposite effects on expression. If the mutations generate new or higher affinity binding sites for a particular factor, it is possible that the context of adjacent transcription factor binding events could influence the recruitment of activating or repressive complexes. Alternatively, the evolution of a strong binding site may cause other factors previously bound to the region to be displaced. If a cooperative interaction between two factors is evolving, then the intermediate step in which just one factor is present may cause a reduction in activity, simply due the displacement of a protein that was previously contributing to the activity. Future elucidation of the transcription factors that comprise the *Nep1* optic lobe enhancer will allow us to distinguish these and other competing models of how regulatory mutations interact cooperatively and antagonistically.

### (c) Co-option of existing activities: opportunities for novelty and pleiotropy

Previous work has posited several mechanisms by which new regulatory activities may arise [36,42–44]. Although evidence exists for several of the possible mechanisms [9,30,43,45], the *Nep1* optical lobe enhancer represents an example of co-option of a pre-existing regulatory activity [36]. The reuse of pre-existing architecture through co-option offers many advantages over the stepwise evolution of enhancers de novo by allowing complex regulatory schemes to be built in fewer evolutionary steps. However, in both adaptive and neutral evolutionary contexts, it also poses distinct challenges. New activities that evolve in the middle of existing enhancers run the risk of altering the activity of those enhancers, making the evolutionary path susceptible to pleiotropic effects. This characteristic can be seen as a structural constraint that is unique to the origination of new enhancers by co-option. Our study uncovered evidence that intermediates during the evolution of the optic lobe enhancer drive differing levels of expression in tissue regulated by a pre-existing overlapping enhancer. While it is uncertain whether the full regulatory region is able to buffer the pleiotropic effects incurred by the evolution of optic lobe activity, our data illustrate a constraint that may govern the modification of a co-opted enhancer.

### (d) The pleiotropic effects of ectopic expression

Mis-expression of a gene can be catastrophic. This is evidenced by the widespread incidence of such effects in genetic disorders and disease [46]. Overexpression is a widely used tool in genetic research, precisely because it often produces phenotypes that are not visible during loss of function studies [47]. Evolutionary paths that lead to ectopic expression will be instantly evaluated by selection as these routes would convey dominant effects on expression. Thus, the *Nep1* case raises the possibility that the path of enhancer evolution may be commonly restricted to paths which eliminate ectopic expression.

Enhancers harbour an enormous potential to generate ectopic activities. The transcription factors that activate enhancers are deployed repeatedly in many locations during development [48]. Although the binding of an upstream transcription factor could lead to activation in a multitude of tissues, ‘combinatorial logic’ is thought to restrict an enhancer's activity to one or a few developmental contexts [21]. Nevertheless, two recent examples demonstrate how rearrangement of existing binding sites in an enhancer can generate novel ectopic activities [22,49]. In a striking example of an enhancer's potential to generate ectopic activity, Liu & Posakony [49] demonstrated how the same combination of transcription factors mediate expression of distinct target genes in two separate Notch-responsive settings during *Drosophila* development. A simple shift in the position of a POU-HD binding site within the *Enhancer of split m*α** enhancer was sufficient to cause weak expression in additional Notch-responsive settings. Thus, although a combination of binding sites may generate expression in multiple tissues, their relative positioning and orientation may be instrumental in controlling the enhancer's specificity. Our results resonate with these studies in that the order in which mutations are introduced can influence the degree to which expression is observed in ectopic locations.
